# Appendiceal Mucinous Neoplasm: A Case of an Incidental Finding

**DOI:** 10.7759/cureus.59540

**Published:** 2024-05-02

**Authors:** Patricia Ward, Taylor Collignon, Taylor Florio, Shivon Barwari, Glenn Miller

**Affiliations:** 1 General Surgery, St. George's University School of Medicine, St. George's, GRD; 2 General Surgery, Larkin Community Hospital Palm Springs Campus, Hialeah, USA; 3 General Surgery, Lake Erie College of Osteopathic Medicine, Bradenton, USA; 4 Plastic and Reconstructive Surgery, Larkin Community Hospital Palm Springs Campus, Hialeah, USA; 5 Urology, Larkin Community Hospital Palm Springs Campus, Hialeah, USA

**Keywords:** appendiceal, low grade, high grade, neoplasm, mucinous

## Abstract

Appendiceal mucinous neoplasms (AMNs) are uncommon gastrointestinal tumors characterized by mucus accumulation in the appendix. Patients may complain of acute appendicitis-like symptoms with other alarming features, but approximately half of the cases of AMNs are found incidentally on imaging. Early diagnosis and management of these neoplasms are important to prevent malignant progression and complications such as bowel obstruction and pseudomyxoma peritonei. We report a case of a 28-year-old female who initially presented with vomiting and acute left lower abdominal pain radiating to the left flank. Computed tomography (CT) revealed a 1.5 mm stone in the left ureteral vesicular junction and a 2.3 x 2.4 x 5.2 cm cystic tubular mass at the base of the cecum, suspicious of an appendiceal mucocele. An elective laparoscopic appendectomy was performed on this admission, which was converted to a right hemicolectomy due to the pathologic finding of a focally high-grade AMN on intraoperative frozen specimen pathology. This report aims to provide an example of a case of an incidental AMN and how it was diagnosed and managed surgically.

AMNs are rare tumors that originate from the appendix and can pose diagnostic and therapeutic challenges due to their diverse clinical presentations and variable histopathological features. The majority of cases of AMNs are discovered in middle-aged individuals (40-50 years of age) after an appendectomy is performed and examined by pathology. This case report aims to describe a rare presentation of a 28-year-old female patient with an incidental finding of AMN on a CT scan of the abdomen while being worked up for suspected nephrolithiasis. We will provide a comprehensive overview of a unique presentation of AMN, highlighting its clinical manifestations, diagnostic approach, and management strategies.

We present the case of a 28-year-old female patient who presented to the emergency department with complaints of acute left lower quadrant abdominal pain radiating to the left flank and vomiting. After an initial assessment and workup, which included lab investigations and imaging, a diagnosis of unilateral hydronephrosis due to a calculus of the ureterovesical junction was made. However, there was also suspicion of an appendiceal mucocele, as evidenced by a CT scan of the abdomen and pelvis. On admission day one, under the care and management of the urology team, she passed the stone with complete resolution of the presenting symptoms. On hospital day two, she underwent an elective laparoscopic appendectomy followed by a right hemicolectomy due to findings of high-grade mucinous neoplasm on the resected frozen specimen near the base of the appendix. AMN was an incidental finding based on CT imaging and macroscopic findings, which was later confirmed by histopathological assessment and report.

## Introduction

Appendiceal mucinous neoplasms (AMNs) are a group of primary appendiceal tumors that are rare, only accounting for 0.4%-1% of gastrointestinal cancers in the United States [1]. AMNs develop as cystic tumors with neoplastic adenomatous growth and excessive mucus accumulation, leading to dilation of the lumen of the appendix. The classification of AMNs is a topic of controversy, and many arrangements have been proposed [2]. According to the World Health Organization 2019 classification, the main categories of AMNs include mucinous adenoma, low-grade AMN (LAMN), high-grade AMN (HAMN), mucinous adenocarcinoma, undifferentiated carcinoma, and goblet cell adenocarcinoma [3].

Benign adenomas solely invade the mucosa, whereas invasive adenocarcinomas are characterized by penetration and invasion into the muscularis mucosa or beyond. Histologically, low-grade tumors are well-differentiated with minimal cell dysplasia, and high-grade tumors are poorly differentiated with severe cell dysplasia. Clinically, AMNs can be difficult to diagnose, as half of the cases are asymptomatic and discovered incidentally. If symptomatic, patients often report vague symptoms resembling acute appendicitis, including abdominal pain, nausea, and vomiting. Other alarming symptoms include a palpable mass, intussusception, obstruction, and weight loss [1,4]. Pseudomyxoma peritonei (PMP) is a feared complication of AMNs, which is characterized by the rupture of a mucocele and subsequent mucus dissemination into the peritoneum [5,6]. The occurrence of PMP due to AMN is not fully known, but this complication is estimated to have an incidence of 1 to 2 per million per year [7,8]. An open surgical approach is typically favored to minimize the risk of PMP; however, laparoscopic surgery can be performed if diagnostic criteria are met [9]. Early detection and diagnosis of AMNs is critical to determine surgical strategies, decrease the risk of developing PMP, and improve overall patient outcomes [5]. Here, we present a rare case of focally high-grade LAMN found incidentally in a 28-year-old female patient who initially presented with renal stone symptomatology. We present this case to highlight the need to consider AMNs as a differential diagnosis in patients with nonspecific symptoms or suspicious findings on CT scans. The management of these neoplasms should be determined based on preoperative diagnosis, histological results, and proper staging in order to prevent patient harm and improve clinical outcomes.

## Case presentation

A 28-year-old Hispanic female presented to the emergency department (ED) at Larkin Community Hospital in Hialeah, Florida, in August of 2023 with complaints of left lower quadrant abdominal pain radiating to the left flank with associated vomiting. She had a past medical history of fatty liver disease and a past surgical history of a cesarean section in 2021. She denied any known allergies and did not take any at-home medications. She has never smoked and has denied alcohol and illicit drug use. She denied any family history of cancer or blood disorders. On physical examination, she was hemodynamically stable and afebrile. She had tenderness to palpation diffusely over the abdomen and bilateral flanks.

**Figure 1:**
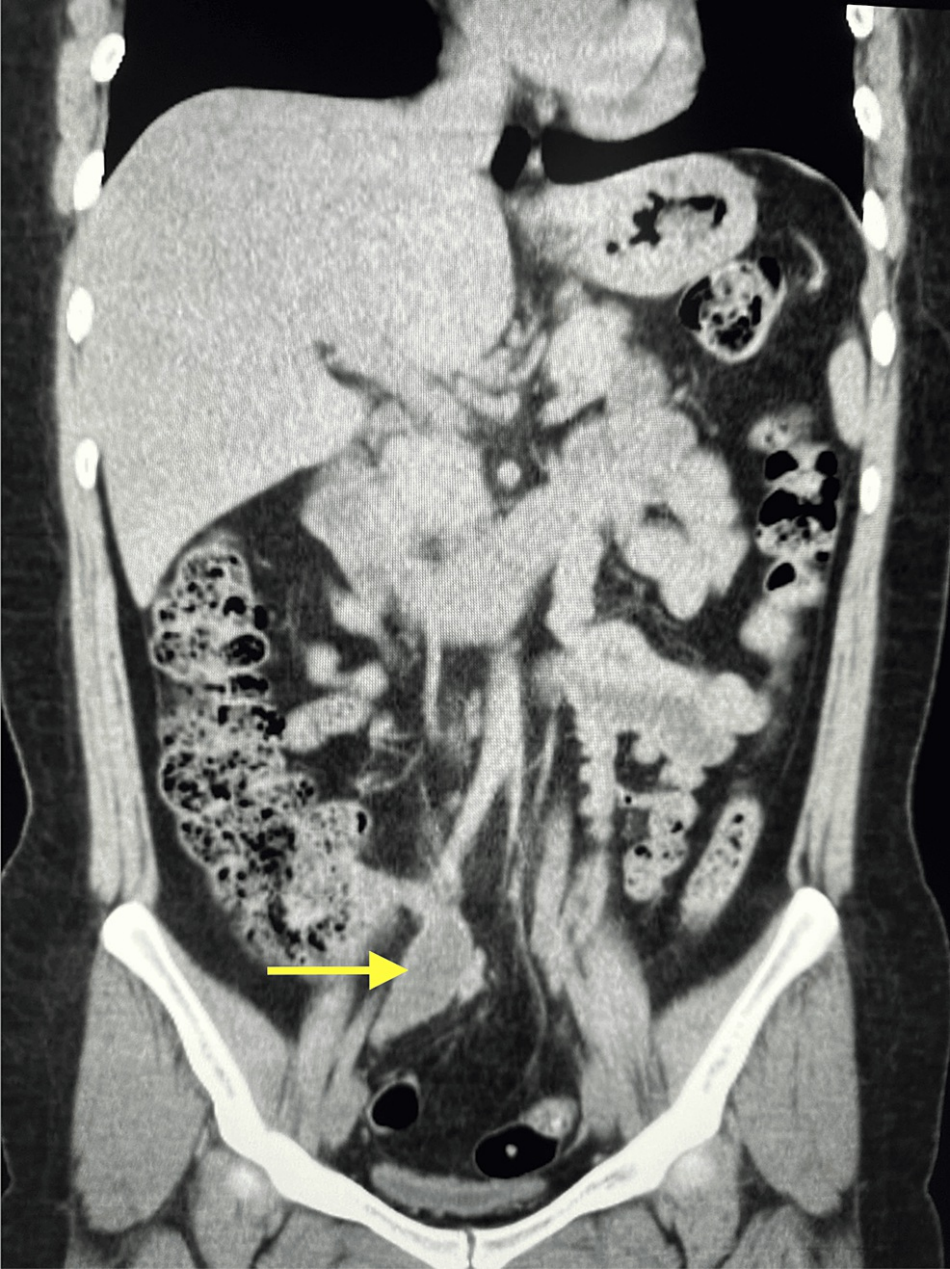
In this coronal CT scan of the abdomen, the yellow arrow points to a 2.3 x 2.4 x 5.2 cm well-circumscribed cystic tubular mass contiguous with the base of the cecum with curvilinear mural calcification suspicious for AMN. CT: computed tomography; AMN: appendiceal mucinous neoplasm

**Figure 2:**
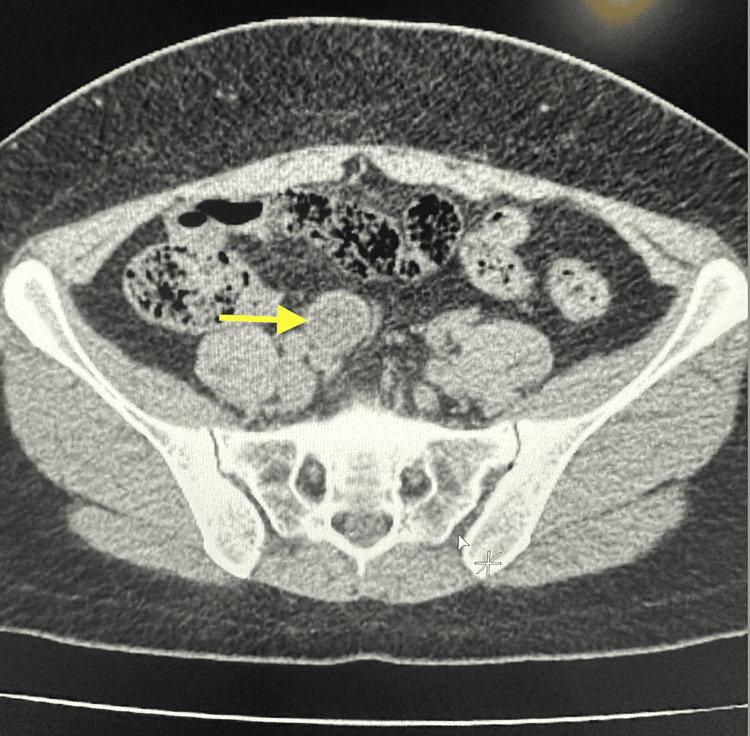
In this axial view CT scan of the abdomen, the yellow arrow is pointing to a 2.3 x 2.4 x 5.2 cm well-circumscribed cystic tubular mass contiguous with the base of the cecum with curvilinear mural calcification suspicious for AMN. CT: computed tomography; AMN: appendiceal mucinous neoplasm

The initial workup showed unremarkable findings, including WBC (8,200/μL), hemoglobin (Hgb) (13.5 g/dL), platelet (PLT) (335), creatinine (Cr) (0.69), glomerular filtration rate (GFR) (103), and CA 19-9 (9 U/mL), all within the normal range (Table 1). CA-125 was elevated (76.9 ng/mL). A non-contrast computed tomography (CT) of the abdomen demonstrated a 1.5 mm stone in the left ureteral vesicular junction (UVJ) with mild hydroureteronephrosis, edema, and inflammation of the left kidney. Due to a lack of contrast, the study was limited, and pyelonephritis could not be ruled out. More notably, the coronal and axial CT scans identified fecalization of the distal ileum with no significant pericolonic fat stranding and a 2.3 x 2.4 x 5.2 cm well-circumscribed cystic tubular mass contiguous with the base of the cecum with curvilinear mural calcification favoring an appendiceal mucocele, as seen in Figures 1, 2.

**Table 1:** Initial workup CBC: complete blood count; WBC: white blood cell; CMP: comprehensive metabolic panel; GFR: glomerular filtration rate

CBC	Reference	Admission
WBC	4.40-10.50 x 10^3^/µL	8.2
Hemoglobin	12.6-16.7 g/dL	13.5
Platelet count	139-361 x 10^3^/µL	335
CMP		
Creatinine	0.6-1.20	0.69
GFR	90-120 mL/min/1.73 m^2^	103
Biomarkers		
CA 19-9	0-37 U/mL	9
CA-125	0-35 U/mL	76.9

At this time, a diagnosis of UVJ calculus with hydronephrosis was made, and there was suspicion of an appendiceal mucocele. Urology was consulted to manage the renal stone on the left UVJ and possible pyelonephritis. The patient was started on tamsulosin and empiric antibiotic therapy with Flagyl and levofloxacin as per the infectious disease (ID) team recommendations. On admission day two, the patient passed the stone overnight with symptom resolution the next day and had no signs of fever or leukocytosis.

General surgery was consulted regarding the incidental appendix findings on the CT, and it recommended the patient have an elective laparoscopic appendectomy to rule out a neoplasm further. After receiving informed consent on admission day three, an elective laparoscopic appendectomy was performed.

During the laparoscopic appendectomy, the appendix was noted to be fairly large, hyperemic, edematous, and showed signs of angiogenesis. At this time, the pathology of the appendix was unclear, and care was taken to prevent spillage and subsequent peritoneal metastasis. Lateral retraction was used on the peritoneal attachments, the appendix was elevated, and dissection was performed through the mesoappendix at the base of the appendix near the cecum. When a clear window was visualized, the stapler was used to transect, followed by the stapling of the mesoappendix. A frozen specimen was obtained, and the specimen was identified as a high-grade dysplasia mucinous neoplasm by the pathologist. Based on the current literature and guidelines, a right hemicolectomy for oncologic resection was indicated. During laparotomy, no swollen lymph nodes were identified while running the small bowel, and no masses were visualized on the liver. A 7 x 2 cm in diameter vermiform appendix with mesoappendix was sent to pathology. The lumen of the appendix was dilated up to 1.8 cm and occupied by a thick mucous material. The surface was inked black. A 7 x 3.5 cm in diameter terminal ileum, 8 x 6 cm cecum, and 16 x 7 cm ascending colon with 30 x 6 cm in depth attached to the adipose tissue was also sent to pathology. Grossly, the serosa of the colonic sections appeared smooth and glistening, while the mucosa was slightly hyperemic.

**Figure 3:**
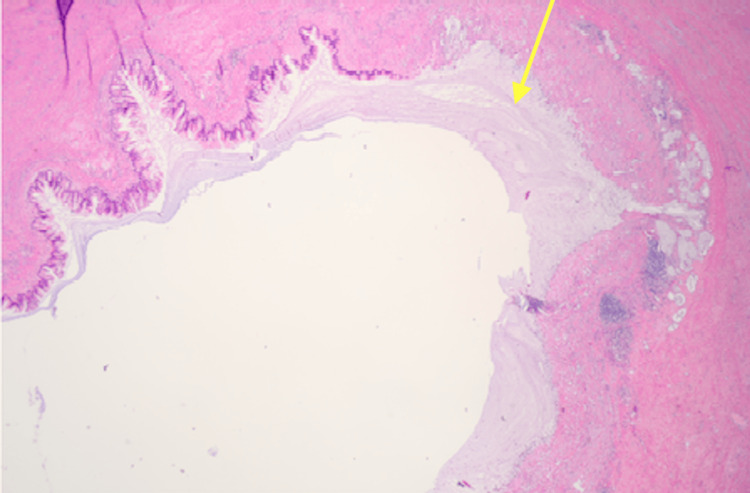
H&E stain at 2× magnification of the specimen sent to pathology consistent with mucinous neoplasm H&E: hematoxylin and eosin

**Figure 4:**
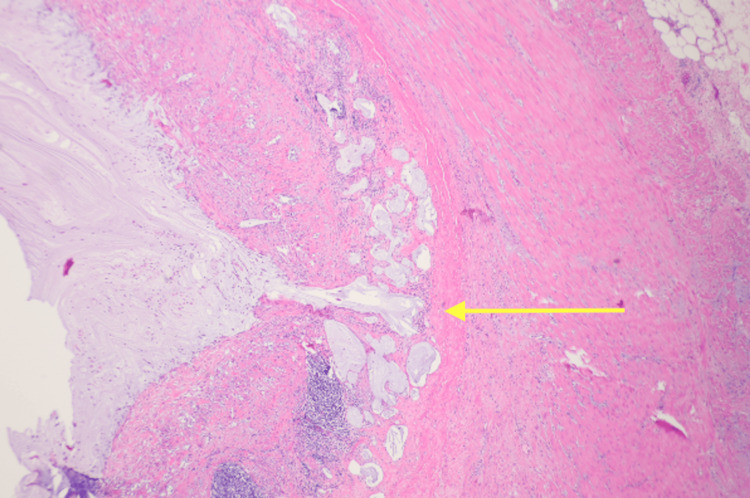
H&E stain at 10× magnification of the specimen sent to pathology showing invasion of mucin into the muscularis propria H&E: hematoxylin and eosin

**Figure 5:**
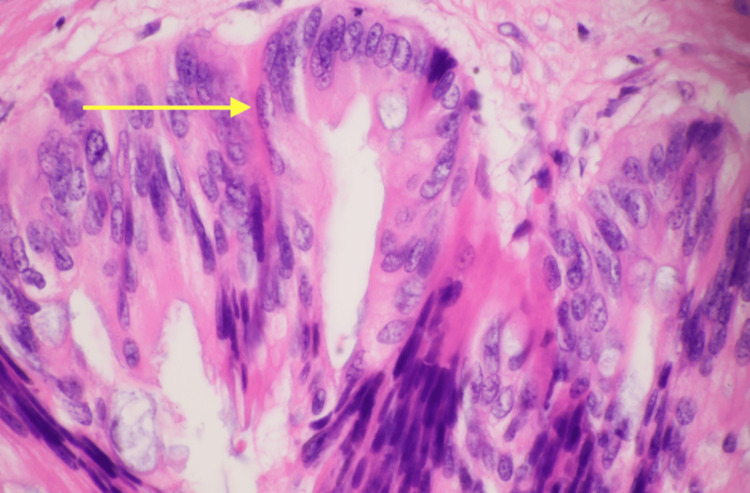
H&E stain at 60× magnification of the specimen sent to pathology showing focal high-grade features of the AMN specimen H&E: hematoxylin and eosin; AMN: appendiceal mucinous neoplasm

The pathological report indicated the mass to be a low-grade AMN (Figure 3) with focally high-grade features (Figure 4) and a benign ileocolonic segment s/p appendectomy. Furthermore, the report identified acellular mucin invading the muscularis propria (Figure 5) with all margins negative for non-invasive tumor, and 13 regional lymph nodes tested negative for tumor. The postoperative pathological tumor-node-metastasis (pTNM) classification concluded a staging of pTisN0; pM was not applicable in this case as it could not be determined from the submitted specimen.

The patient did well postoperatively. She was seen and evaluated by hematology/oncology for the finding of a focally high-grade, low-grade AMN before discharge. At this time, laboratory investigations of cancer markers were evaluated and showed an elevated CA-125 at 76.9 and an unremarkable CA 19-9 at 9. Finally, she was discharged on day six of admission and postoperative day three s/p appendectomy and right hemicolectomy, with plans in place to be followed up in an outpatient setting by general surgery and hematology/oncology.

## Discussion

AMNs are a diverse group of neoplasms that present diagnostic challenges due to their complex nature and unconventional clinical presentations. These range from simple mucoceles to complicated malignancies, and care must be taken to prevent malignant progression, metastasis, or abdominopelvic complications such as obstruction or rupture [6,10]. Mucoceles generally affect women more than men, and the peak incidence is after 50 years of age [5]. The risk factors for malignant mucoceles are not fully elucidated at this time, but there is an association of appendiceal adenocarcinomas with chronic ulcerative colitis and colonic neoplasia [1]. A previous study found that low-grade AMN had a three-year survival rate of 100% and a five-year survival rate of 86%, whereas malignant AMN's three-year survival rate was 90% and a five-year survival rate of 44% [2]. The patient presented in this case report was younger than the peak onset for mucoceles and did not have risk factors such as chronic ulcerative colitis or colonic neoplasia.

AMNs may present similarly to numerous abdominal cystic findings, so it is critical to consider them in the list of differentials alongside appendicitis, lymphoma, colorectal cancer, or gynecologic cancers and tumors. In this case, the patient presented with symptoms of left lower abdominal pain radiating to the left flank and nausea. The mucinous neoplasm was found incidentally on a CT scan. Her symptoms were relieved when the renal stone passed; therefore, her presenting symptoms were likely not due to the appendiceal neoplasm. Had she not developed her renal pathology, her appendiceal neoplasm would have likely been asymptomatic until it grew large enough to cause a mass effect or metastasize. Generally, it is difficult to distinguish the cellular composition and malignant potential of AMNs based on imaging alone, but characteristic findings such as septations and the presence of an enhancing solid mass raise suspicion for cancer in the majority of cases [11,12]. Here, the imaging showed a well-circumscribed cystic tubular mass contiguous with the base of the cecum with curvilinear mural calcification, which raised suspicion of an appendiceal mucocele. At that point, malignancy could not be ruled out, and surgical exploration was warranted. Therefore, although imaging techniques are useful for preliminary diagnosis and complication prevention, surgical removal and pathological examination are required for diagnostic confirmation [12].

Recent findings suggest that LAMN is likely a precursor to HAMN, and the two pathologies share high rates of similar tumorigenic mutations, including *KRAS* and *GNAS* [13]. It is established that these may coexist in the same lesion, i.e., the presence of HAMN may be found focally among a background of LAMN, as seen in this case [12,14]. Histopathologic findings of HAMN, positive margins, or tumors measuring 2 cm or greater are indications for right hemicolectomy during surgical excision of AMN [12]. In this case, a high-grade dysplasia mucinous neoplasm was identified by the pathologist, and the general surgery team proceeded with a right hemicolectomy according to current guidelines. We report a case of AMN found incidentally on a CT scan and highlight the need to consider this pathology in the setting of cystic abdominal masses presenting with nonspecific symptoms. AMNs and other cystic mass ruptures can place patients at risk of developing PMP. For this reason, a fine needle biopsy should be avoided, and surgical caution should be advised to prevent neoplasm puncture and dissemination. Physician judgment is primarily relied on when it comes to removing additional structures in the context of large tumors or high-grade pathologies. Further research is necessary to outline standardized management for these tumors and to remove the ambiguity surrounding proper treatment.

## Conclusions

In conclusion, AMNs are a rare pathology with insidious onset and asymptomatic presentation or nonspecific manifestations. Here, we present a case of focally high-grade LAMN found incidentally on a CT scan in a 28-year-old female patient who initially presented with renal stone symptomatology. We present this case to highlight the need to consider AMNs as a differential diagnosis in patients with nonspecific symptoms or abnormal findings on CT scans. This case report provides valuable insights into the diagnostic and therapeutic considerations for AMNs as an incidental finding on CT of the abdomen. The rarity of these tumors underscores the need for a comprehensive understanding of their clinical behavior and appropriate management strategies. Sharing experiences and insights from individual cases contributes to the collective knowledge that informs evidence-based practices in the care of patients with AMNs.

## References

[1] Tirumani SH, Fraser-Hill M, Auer R, Shabana W, Walsh C, Lee F, Ryan JG (2013). Mucinous neoplasms of the appendix: a current comprehensive clinicopathologic and imaging review. Cancer Imaging.

[2] Misdraji J (2010). Appendiceal mucinous neoplasms: controversial issues. Arch Pathol Lab Med.

[3] Koç C, Akbulut S, Akatlı AN, Türkmen Şamdancı E, Tuncer A, Yılmaz S (2020). Nomenclature of appendiceal mucinous lesions according to the 2019 WHO Classification of Tumors of the Digestive System. Turk J Gastroenterol.

[4] Liapis SC, Perivoliotis K, Psarianos K (2023). A giant low-grade appendiceal mucinous neoplasm (LAMN) presenting as ileocecal intussusception: a case report. J Surg Case Rep.

[5] Spyropoulos C, Rentis A, Alexaki E, Triantafillidis JK, Vagianos C (2014). Appendiceal mucocele and pseudomyxoma peritonei; the clinical boundaries of a subtle disease. Am J Case Rep.

[6] Wang AS, Ismael HN, Parikh J, Modesto VL (2022). Low-grade appendiceal mucinous neoplasm: a case series. Cureus.

[7] Smeenk RM, van Velthuysen ML, Verwaal VJ, Zoetmulder FA (2008). Appendiceal neoplasms and pseudomyxoma peritonei: a population based study. Eur J Surg Oncol.

[8] Mittal R, Chandramohan A, Moran B (2017). Pseudomyxoma peritonei: natural history and treatment. Int J Hyperthermia.

[9] Orcutt ST, Anaya DA, Malafa M (2017). Minimally invasive appendectomy for resection of appendiceal mucocele: case series and review of the literature. Int J Surg Case Rep.

[10] Tajima T, Tajiri T, Mukai M (2018). Single-center analysis of appendiceal neoplasms. Oncol Lett.

[11] Lu A, Cho J, Vazmitzel M, Layfield L, Staveley-O'Carroll K, Gaballah A, Rao D (2021). High-grade appendiceal mucinous neoplasm presenting as a giant appendiceal mucocele. Radiol Case Rep.

[12] Shaib WL, Assi R, Shamseddine A (2017). Appendiceal mucinous neoplasms: diagnosis and management. Oncologist.

[13] Liao X, Vavinskaya V, Sun K (2020). Mutation profile of high-grade appendiceal mucinous neoplasm. Histopathology.

[14] Misdraji J, Yantiss RK, Graeme-Cook FM, Balis UJ, Young RH (2003). Appendiceal mucinous neoplasms: a clinicopathologic analysis of 107 cases. Am J Surg Pathol.

